# A New Composite Material with Environmental Implications for Sustainable Agriculture

**DOI:** 10.3390/ma16196440

**Published:** 2023-09-27

**Authors:** Viorica Ghisman, Puiu Lucian Georgescu, Georgiana Ghisman, Daniela Laura Buruiana

**Affiliations:** 1 Interdisciplinary Research Centre in the Field of Eco-Nano Technology and Advance Materials CC-ITI, Faculty of Engineering, “Dunarea de Jos” University of Galati, 47 Domneasca, 800008 Galati, Romania; viorica.ghisman@ugal.ro (V.G.); georgiana.ghisman@ugal.ro (G.G.); 2 European Center of Excellence for the Environment, Faculty of Sciences and Environment, University of Galati, 800001 Galati, Romania; lucian.georgescu@ugal.ro

**Keywords:** composite material, sewage sludge, dump slag, soil

## Abstract

Sewage sludge, also referred to as biosolids, consists of the by-products of wastewater treatment, which are a mixture of the water and organic and inorganic materials eliminated from wastewater sourced from domestic sewage industries during physical, biological, and/or chemical treatments. Biosolids are nutrient-rich organic materials resulting from the treatment of domestic wastewater in treatment plants. Sewage sludge can be considered a significant biological resource for sustainable agriculture. A new composite has been made consisting of soil, sewage sludge collected from the Galati Wastewater Treatment Plant, and slag from the Galati Steel Plant. This study aimed to investigate the structural, morphological, and chemical parameters of this composite through FTIR, SEM-EDX analysis, and XRF analysis. The samples had values of potential toxic elements that were within normal limits according to Directive 86/278/CEE, and, in terms of the iron-to-calcium ratio (I/C), all samples were of a low grade. This is the first time that slag has been added in a sewage sludge–soil combination, which can be an effective fertilizer replacement. Sewage sludge contains substantial amounts of organic matter, and slag reduces the contents of potentially toxic elements. In addition to these attributes, they may provide an opportunity for the beneficial re-use of sewage sludge and slag as resources in agriculture.

## 1. Introduction

Nowadays, the excessive use of conventional fertilizers combined with the problems of climate change, global warming, urbanization, the unbalanced use of resources, and environmental problems visibly affect the qualities of soil. Every year, large areas of soil are at risk of desertification [1]. Desertification, which is the greatest environmental challenge of our time, means a decline in soil fertility. Two of the objectives of the Urban Waste and Sewage Sludge Management Plan (2016–2020) for Piemonte, Italy, consisted of the reduction and prevention of desertification through the incentive of sewage sludge in agriculture (home composting) and the improvement of water source quality through the promotion of sewage sludge use in agriculture and reductions in biodegradable waste disposal in landfills [2]. Sewage sludge is the final waste obtained from the wastewater treatment process. Urban wastewater consists of impure water released by residential, institutional, commercial, and industrial establishments. The resulting wastewater is also mixed with surface water and additional rainwater. Such water carries all sorts of unwanted components and is, therefore, collected from the sewer and directed to a wastewater treatment plant for purification [3]. Sewage sludge, also referred to as biosolids, consists of the by-products of wastewater treatment, which are a mixture of the water, organic, and inorganic materials eliminated from wastewater that is sourced from domestic sewage industries during physical, biological, and/or chemical treatments. Biosolids are nutrient-rich organic materials that result from the treatment of domestic wastewater in treatment plants [4]. Traditional methods for the disposal of sewage sludge are costly and cause environmental pollution. Investigations into water quality and pollution sources by identifying and predicting the dynamics of the state parameters of a complex aquatic system are essential for the implementation of sustainable management strategies [5]. The use of nanoparticles in polymeric membranes is a way to remove unwanted elements from wastewater streams, as developed by the authors of [6], who showed that this has an important influence on improving water permeation, retention potential, and fouling resistance. The authors of [7,8] affirmed that sewage sludge is a good source of plant nutrients and organic matter, and that it is best to utilize this waste as organic fertilizer due to its high levels of plant nutrients, such as N, K, P, Zn, Fe, Cu, Mn, and other trace elements. An alternative for using the sludge resulting from the processing of urban wastewater is its use as raw matter in the process of cast iron production in blast furnaces [9]. Some researchers regard sewage sludge as stabilized organic solids derived from the biological wastewater treatment process, which can be used in agriculture as a source of waste disposal [10]. Jamil M et al. showed that the addition of domestic sewage sludge raises soil pH, increases organic matter, electrical conductivity, and macro and micronutrients, and as its levels are increased it has beneficial effects on soil properties and the yield of wheat crops P [11]. P. Csattho affirmed that the organic matter content in sludge can improve soil’s chemical, physical, and biological properties, with the assurance of a better culture and aquifer capacity of the soil [12]. Applying sewage sludge to agricultural land is the best method to recycle the nutrients present in it. Therefore, sewage sludge can be considered a significant biological resource for sustainable agriculture; it produces favorable plant yield responses when used as organic fertilizer [13,14]. Another way to increase the crop yield is to pay attention to the correction of soil acidity. Wen et al. [15] showed that applying steel slag onto PTE acidic mining soils effectively increases the soil pH and soil microbial abundance and immobilizes PTE ions, which provide a desirable plant survival climate. In previous studies, the pH of acidic soil was improved by using a mixture formed by slag waste dumped in a landfill and granulated blast furnace slag [16].

The present study aims to show the effects of the content of organic matter in urban sewage sludge resulting from wastewater treatment and of waste slag dumped in a landfill resulting from steel production and their potential as resources in agriculture.

## 2. Materials and Methods

Considering both the positioning of Romania in the hydrographic basin of the Black Sea and the Danube River, as well as the need for environmental protection in these areas, the entire territory of Romania has been declared to be a sensitive area. This decision was made concrete by the fact that agglomerations with more than 10,000 equivalent inhabitants must maintain an infrastructure for wastewater treatment that allows for advanced treatment—especially from the point of view of nitrogen and phosphorus nutrients. From the point of view of the degree of purification, secondary purification from the biological stage is a general rule for agglomerations with less than 10,000 equivalent inhabitants.

The evolution of the degree of connection to the collection and purification systems of the population—depending on the type of purification process applied—leads to an increase in the population that benefits from the wastewater collection services, which leads to the expansion and construction of related infrastructure. Recently, it could be observed that the proportion of collection systems with mechano-biological and tertiary purification has increased in congested areas where the population is over 15,000 equivalent inhabitants, as these stations effectively remove organic matter, phosphorus, and nitrogen compounds.

Mechanical treatment plants remove suspended solids (approx. 40–70%) and biological treatment plants use aerobic and/or anaerobic microorganisms to remove ammonium (approx. 75%), retaining part of the nutrients (approx. 20–30%) and decomposing some organic substances (approx. 50–80%). In 2015, a total volume of wastewater of approximately 1943 million m^3^ was generated, of which 1005 million m^3^ was water from economic activities and 938 million m^3^ was from domestic activities. In 2020, the total volume of wastewater decreased to 1709 million m^3^, of which 925 million m^3^ came from household activities and 784 million m^3^ from economic activities.

This decrease in wastewater from the sphere of economic activities is due to either a reduction in the amount of production, the interruption of economic activities due to the SARS-CoV2 pandemic, or even the bankruptcy of certain enterprises. From the situation provided by the National Institute of Statistics regarding the management of sludge from urban sewage treatment plants for the year 2019 in Romania, there was a total amount of sludge obtained from sewage treatment plants of 230.59 thousand tons of dry matter, reaching almost 55% of the planned value from 2015—a value that is placed at approx. 44% of the 2020 value.

Of the total sludge obtained, 130.02 thousand tons remained stored on specially designed platforms, 43.56 thousand tons were used in agriculture, 12.19 thousand tons were used for composting or other applications, 1.14 thousand tons were incinerated, and the rest of the 43.67 thousand tons were used in other applications.

Romanian legislation in the management of sewage sludge is adjusted to the EU Directives by Government Decision no. 1157 of 13.10.2008, regarding the approval of the Technical Regulation “Soil protection measures in agricultural practices”, where art. 11 stipulates that the sludge from sewage treatment plants can be used in agriculture so that the accumulation of heavy metals in the soil does not lead to exceeding the limit values and so that the concentrations accumulated over 10 years on the same surface are not exceeded. Methods for sludge and soil analysis are provided. Furthermore, the use of sludge is prohibited on:

- pastures or on fodder crops, with a minimum of three weeks before the start of grazing and harvesting of fodder crops;
- lands cultivated with vegetables and fruits during the growing season, except of fruit tree crops;
- soils intended for vegetable and fruit crops, for 10 months before harvesting and during harvesting.

However, this Government Decision does not provide the responsibilities and duties of the interested parties involved in the management of sewage sludge.

### 2.1. Area Description and Sampling

**Figure 1:**
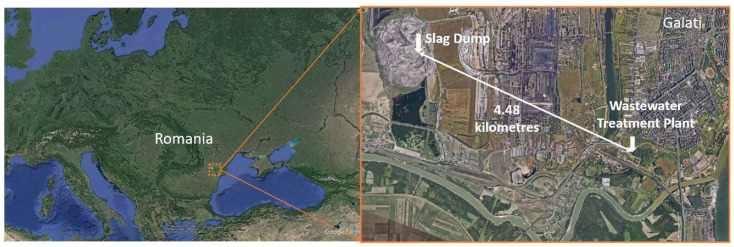
Map of the sampling site location Galati, Romania (Google Earth Pro software 7.3 version).

The sewage system of the municipality of Galati covers an area of 2300 ha in the urban area of the municipality of Galati. The sewage network of the municipality of Galati has a total length of 533.9 km, of which 526.9 km is in the unitary system and 7 km is rainwater. For the evacuation of waste and rainwater, the sewage system has 10 pumping stations. The Galati Wastewater Treatment Plant (Figure 1) has a capacity of about 360,000 p.e., it is equipped with a mechanical step for treating domestic wastewater as well as a section for treating the associated sludge in anaerobic mode. The sludge is stabilized in a 6000 cubic meter anaerobic digester with a solid retention time of 18 days for a 60% reduction in organic material content and biogas generation. Through fermentation, the minimum standard of stabilized sludge is ensured—as stated in the European Union legislation that refers to the storage of sludge at the waste ramp, the sludge can also be used as an inferior fertilizer in agriculture.

The waste slag was collected from the largest Integrated Steel Company of Galati Slag Dump (Figure 1). The topsoil (0–0.25 m) was taken from a commune (Tulucesti) in Galati County in Romania (no specific authorizations were required for taking soil samples in this location, and the study area did not involve endangered or protected species).

Three types of samples were prepared with different content, such as sewage sludge 100% (Sample 1), sewage sludge:soil 50%:50% (Sample 2) and sewage sludge:soil:slag 25%:50%:25% (Sample 3).

### 2.2. Characterization Techniques

The well-established technique for quantitative determination is FTIR–attenuated total reflection (FTIR–ATR)—a method of recording the attenuated total reflection technique. Fourier transform infrared (FTIR) spectra of the slag samples were recorded using an IRSpirit-T FT-IR Spectrometer (Shimadzu, Tokyo, Japan) equipped with a built-in ATR accessory-type QATR-S (Shimadzu, Tokyo, Japan), DLATGS detector, and KBr beam splitter. The scan range was set at 400–4000 cm^−1^ with a resolution of 2 cm^−1^ and the number of scans was 45. The reference taken was air for the background spectrum before each sample and the ATR plate was cleaned with ethyl alcohol solution after each spectrum. A background spectrum was collected each time and compared to the previous background spectrum to verify that no residue from the previous sample remained. The FT-IR spectrometer was placed in a temperature-controlled (21 °C), air-conditioned chamber.

The morphology and elemental composition of the investigated sewage sludge samples were examined by scanning electron microscopy coupled with energy dispersive X-ray (SEM/EDX) spectroscopy using a FEI Q 200 MICROSCOPE (FEI Company, Hillsboro, OR, USA) in low vacuum. Before examination, the samples were coated with 4 nm-thick conducting layer of Au using a SPI-Module sputter coater (SPI Module™ Supplies, West Chester, PA, USA) system.

The sewage sludge samples were characterized by X-ray Fluorescence (XRF; Vanta V Model VCR-CCC-A3-E, Olympus, Center Valley, PA, USA) for the quantitative determination of major and trace element concentrations in samples using a calibration with matrix-matched standards geo method.

## 3. Results and Discussion

### 3.1. Structural and Morphological Characterization

**Figure 2:**
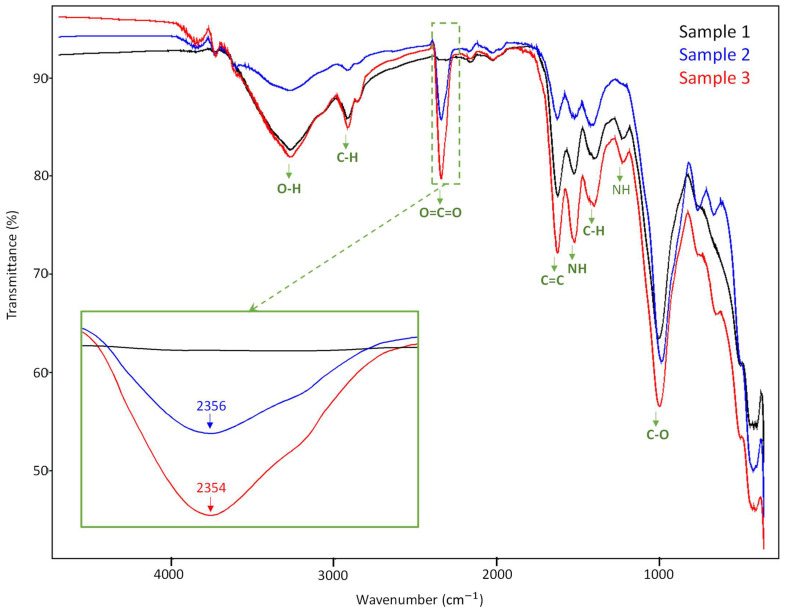
FTIR spectra of sewage sludge (Sample 1), sewage sludge–soil (Sample 2), and sewage sludge–soil–slag (Sample 3).

The FTIR spectra of the sewage sludge (Sample 1), sewage sludge:soil (Sample 2), and sewage sludge:soil:slag (Sample 3) is presented in Figure 2.

By analyzing the spectrum (detailed figure) in the range of 2300–2400 cm^−1^, it can be found that there were obvious absorption peaks in the spectrum only of samples where the sewage sludge was in combination with soil (Sample 2) or with soil and slag (Sample 3). The sewage sludge sample showed characteristic absorption bands at 3746, 3277, 2924, 2359, 2170, 2030, 1636, 1534, 1410, 1234, 1015, 516, and 445 cm^−1^. The mid-intense bands at 3746 and 3277 cm^−1^ were attributed to the stretching vibration of the hydroxyl group originating from the weakly absorbed water molecules on the sewage sludge. The characteristic absorption band at 2924 cm^−1^ was attributed to aliphatic methylene groups and assigned to fats and lipids. Lipids are an important fraction of sewage sludge that can influence the water retention capacity of amended soils, their structural stability, and the biodegradation–humification balance in soils [17]. Two broad bands at 1534 and 1636 cm^−1^ were of protein origin—Amide II and Amide I, respectively. The main absorbance in the FT-IR spectra of the sewage sludge at 1015 cm^−1^ was assigned to C–O stretching in polysaccharides or polysaccharide-like substances, the Si–O of silicate impurities, and clay minerals that were possibly in a complex with humic acids [18]. Observed at 2924 cm^−1^ at medium intensity, duplet band reflected alkyl chains (polyalcohol, saccharides, and fats), referring to the stretching vibration of C–H bonds. The band at 2359 cm^−1^ was attributed to ammonium sulphate. The hemicellulose fraction contained noticeable amounts of polysaccharides and could have contributed to the FTIR spectrum via the bands identified at 1410 and 1234 cm^–1^, which designated the methyl C–H wagging vibrations and carbonyl absorbance in the pectic polysaccharide substances. The combination of sewage sludge with soil (Sample 2) showed characteristic absorption bands at 3865, 3746, 3619, 3279, 2924, 23456, 2179, 2035, 1636, 1542, 1422, 997, 778, 676, 510, and 430 cm^−1^. The bands at 3865, 3746, 3619, and 3279 cm^−1^ were attributed to the stretching vibrations of the O-H bond, the band at 2924 cm^−1^ was attributed to the CH stretching vibration specific to an alkane group, the band at 2356 cm^−1^ was attributed to the O= stretching vibration C=O specific to carbon dioxide, and the band at 2035 cm^−1^ was specific to O=C=O stretching vibrations. The bands at 1636 cm^−1^ and 1542 cm^−1^ were attributed to C=C stretching vibrations due to the alkene group. Bands assigned to C=C bending vibrations were at 1422, 997, 778, and 676 cm^−1^. The band at 510 cm^−1^ was specific to Cl stretching vibrations and the band at 430 cm^−1^ was attributed to CH bending vibrations. When the sewage sludge was combined with soil and slag (Sample 3), the characteristic bands at 3859, 3737, 3277, 2923, 2863, 2354, 2172, 2032, 1636, 1534, 1420, 1233, 1012, 776, 659, 511, 452, and 417 cm^−1^ were shown. The characteristic absorption bands at 3859, 3737, 3277, 2923, and 2863 cm^−1^ were assigned to OH stretching vibrations, the band at 2354 cm^−1^ was assigned to a CN stretching vibration specific to the alkene group, the band at 2172 cm^−1^ was assigned to the vibrations of thiocyanate-specific S-C≡N stretching, the band at 2032 cm^−1^ was assigned to the N=C=S stretching vibration, and the band at 1636 cm^−1^ was assigned to the C=C stretching vibration. The 1534 cm^−1^ band was not assigned to any vibration because it was a nitro compound. The band at 1420 cm^−1^ was assigned to OH stretching vibrations, the band at 1233 cm^−1^ and 1012 cm^−1^ was assigned to CO stretching vibrations, the band at 659 cm^−1^ was assigned to a C-Br stretching vibration, and the 511 cm^−1^ band was attributed to Cl stretching vibrations. The group of peaks at 452 cm^−1^ and 417 cm^−1^ were attributed to CH bending vibrations. One can notice that the absorption bands of the sewage sludge–soil sample (Sample 2) showed higher values when compared to that of the sewage sludge sample (Sample 1), and there were lower values for the combination of sewage sludge–soil–slag (Sample 3). As can be observed, when the slag was added (Sample 3), the intensity of the absorption peaks increased, which could provide important evidence of the chemical interactions between the three components—respectively, sewage sludge, soil, and slag.

**Figure 3:**
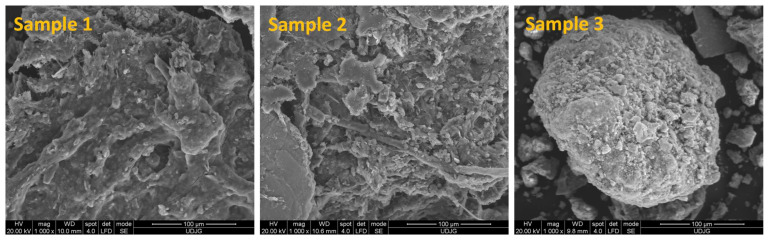
SEM images of sewage sludge (Sample 1), sewage sludge–soil (Sample 2), and sewage sludge–soil–slag (Sample 3).

The SEM micrographs of the sewage sludge samples (Sample 1–3) are presented in Figure 3. One can see the characteristic morphology—the sizes and the forms—of the sew-age sludge samples.

In the SEM images of Sample 1, you can see the organic formations represented by the sharp peaks that were due to the internal symmetry of the sample. In Sample 2, the presence of rounded formations and different morphologies such as spheres, rods, and plates were observed. The sewage sludge–soil–slag sample displayed a rough and uneven surface of aggregated particles (average diameter of a few microns) with different morphologies such as spheres, rods, and boards specific to metallurgical slag [16].

### 3.2. Chemical Analysis

**Figure 4:**
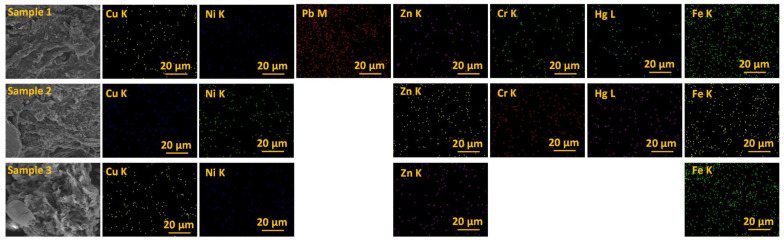
EDX elemental map of sewage sludge (Sample 1), sewage sludge–soil (Sample 2), and sewage sludge–soil–slag (Sample 3).

Figure 4 presents the EDX elemental analysis of selected chemical elements such as copper, nickel, lead, zinc, chromium, mercury, and iron present in sewage sludge (Sample 1), sewage sludge–soil (Sample 2), and the combination of sewage sludge–soil–slag (Sample 3).

The chemical elements of interest regarding the sewage sludge used in agriculture [19] are presented in EDX mapping (Figure 4). As can be seen, the lead did not appear in the sewage sludge–soil and sewage sludge–soil–slag samples, in contrast to the sewage sludge sample. Additionally, chromium and mercury were not present in the sewage sludge–soil–slag (Sample 3), in contrast to the other two samples of sewage sludge (Samples 1–2). As previously mentioned, the positive effect of the use of slag in the soil purification sludge compound, regarding the quantities of admissible heavy metals, can be seen. Nutrients are components necessary for the growth and development of plants. It would be easy to consider nutrient at a particular time, but it must be kept in mind that the plant needs nutrients holistically. Plants need 14 essential elements, represented as soil nutrients that are divided into two categories: macronutrients and micronutrients; macronutrients include nitrogen, phosphorus, potassium, sulfur, calcium, and magnesium [20]. From the EDX analysis, all the samples contained the macronutrients beneficial to agriculture, and for plants.

From the EDX spectra, the percentage (At%) of nitrogen content was obtained as 1.10 for Sample 1, 2.74 for Sample 2, and 2.75 for Sample 3—as can be seen in Figure S1. The EDX spectra show a qualitative chemical analysis of the analyzed sewage sludge–soil–slag sample (Sample 3). As can be seen, the content of nitrogen was bigger in the sewage sludge–soil–slag composite (Sample 3), which is beneficial for its use in agriculture. The biomass N content was uncharacteristically low, resulting in a mean microbial biomass C:N ratio of 56:1 for Sample 1 that increased to 20.5:1 for Sample 2 and Sample 3. Despite the C:N ratio, sludge application enhanced the N mineralization potential of the soil. Sludge application somewhat increased the activities of important soil enzymes. Therefore, sewage sludge may be considered an important biological resource for sustainable agriculture. 

The chemical composition of the major elements that comprised the sewage sludge, sewage sludge–soil and sewage sludge–soil–slag samples was determined by XRF analysis. The values, expressed as ppm of chemical elements, are presented in Table 1 and Table 2, the recommended values from Directive 86/278/CEE regarding the usage of sewage sludge in agriculture are presented. As can be seen, the content of the main constituents in all the samples investigated was aluminum, silicon, phosphorus, sulfur, potassium, calcium oxide, titanium, manganese, iron oxide, and potentially toxic elements (PTE) such as arsenic, zinc, and copper. For the sewage sludge sample, higher values of potentially toxic elements (PTE) and a low content of elements compared with the other two samples can be observed. When the soil was added, it could be seen that the values of potentially toxic elements (PTE) decreased compared with sewage sludge sample, but increased the value of low elements, which may indicate the inorganic element contents of the soil. In the case of the sewage sludge–soil–slag sample (Sample 3), the values of the potentially toxic elements (PTE) decreased compared to the other two samples, and the disappearance of the arsenic and yttrium with the addition of the slag component can be observed. The higher content of Ca^2+^ in the new composite material (Sample 3) due to the presence of slag was able to maintain high alkalinity in the soil for a long time in the natural environment. The alkaline pH of slag (Table 3) may contribute to a decrease in the available concentration of heavy metals by reducing metal mobility and bonding metals into more stable fractions. Based on these XRF results, it can be can said that an elimination of potentially toxic elements took place in the sewage sludge–soil, as well as a decrease in the low elements percentage by applying slag.

Table 2 shows that the detected concentrations in the sewage sludge–soil–slag sample were lower than the recommended values from Directive 86/278/CEE on the protection of the environment, and in particular of soil, when sewage sludge is used in agriculture. The purpose of this Directive is to regulate the use of sewage sludge in agriculture in such a way as to prevent harmful effects on soil, vegetation, animals, and man—thereby encouraging the correct use of such sewage sludge to ensure that those limit values are not exceeded.

The elemental oxides were used to calculate the slagging index regarding the Iron to Calcium Ratio (I/C), with the formula Fe_2_O_3_/CaO for the sewage sludge samples. In terms of the iron to calcium ratio (I/C), all samples were in the low grade. The distribution of Polycyclic Aromatic Hydrocarbons (PAH) in Sample 1, Sample 2, and Sample 3 is shown in Table 4. The PAH results of the three samples show that the sum of PAH compounds was within the recommended values of ORDER no. 344/708/2004 [22] on the technical norms regarding the protection of the environment and especially of soils when sewage sludge is used in agriculture. Based on these results, it may recommend that a new composite material consisting of sewage sludge, soil, and slag can be used in agriculture as a fertilizer. 

## 4. Conclusions

There is an increasing interest in the agricultural applications of sewage sludge obtained from wastewater treatment plants due to the possibility of the recycling of valuable components such as organic matter, N, P, and other plant nutrients. Since sewage sludge contains substantial amounts of macronutrients, it can be an effective fertilizer replacement for these important nutrients. 

This is the first time that slag has been added into a sewage sludge–soil combination, which could be an effective fertilizer replacement as sewage sludge contains substantial amounts of organic matter and slag can reduce the content of potential toxic elements. In addition to these attributes, it may provide an opportunity for the beneficial re-use of sewage sludge and slag as a resource in agriculture. The waste slag dumped in a landfill was used in combination with sewage sludge and soil to recover and recycle resources from metallurgical waste and to improve the soil’s properties. From the FTIR spectra of the newly obtained composite, the intensity of the absorption peaks increased compared with the sewage sludge and sewage sludge–soil, which provides important evidence of chemical interactions between the three components—respectively, sewage sludge, soil, and slag. From the EDX analysis, all of the samples contained macronutrients beneficial to agriculture, as well as for plants. The XRF analysis showed that the detected concentrations in the sewage sludge–soil–slag sample were lower than the recommended values in Directive 86/278/CEE on the protection of the environment, and in particular of soil, when sewage sludge is used in agriculture.

From the scope of this research, a highly ambitious and transformative vision was established by finding new opportunities for the sustainable recycling and safe global reuse of sewage sludge and landfill slag waste. In addition to these attributes, it can conserve organic matter and provide an opportunity for the beneficial reuse of both sewage sludge and waste slag dumped in landfill as a resource in agriculture and recovery, rather than their disposal.

## Figures and Tables

**Table 1:** Results of the XRF analysis of sewage sludge (Sample 1), sewage sludge–soil (Sample 2), and sewage sludge–soil–slag (Sample 3).

Elements	Sample 1		Sample 2		Sample 3	
	PPM	+/− 3σ	PPM	+/− 3σ	PPM	+/− 3σ
**Al**	10,700	2700	11,000	3300	13,900	4400
**Si**	38,800	1900	52,900	2700	72,800	4300
**P**	11,350	570	3580	400	4340	580
**S**	10,390	520	4020	440	4250	610
**K**	11,730	470	11,420	540	12,790	770
**CaO**	77,000	2500	54,900	2100	183,500	8400
**Ti**	4700	1000	3700	1100	6000	1800
**Mn**	6590	460	3140	340	8390	830
**Fe_3_O_4_**	51,400	2000	42,500	1900	70,800	3900
**Ni**	-	-	-	-	122	90
**Cu**	333	60	185	53	101	69
**Zn**	928	82	578	69	539	96
**As**	36	18	22	18	-	-
**Rb**	52	11	69	12	61	18
**Sr**	323	21	257	21	246	29
**Y**	13	11	13	13	-	-
**Zr**	76	16	101	18	102	28
**Nb**	15	10	18	11	44	18
**Pb**	25	22	29	21	42	38
**Th**	50	32	75	36	128	59
**U**	27	16	17	17	29	28
**LE**	808,900	6800	836,600	6900	690,000	15,000

**Table 2:** Recommended values of parameters from Directive 86/278/CEE.

Parameters	Recommended Values from Directive 86/278/CEE [19].	Detected Concentrations in Sewage Sludge–Soil–Slag Sample (Sample 3)
**Cd**	20–40	0.0
**Cu**	1.000–1.750	101
**Ni**	300–400	122
**Pb**	750–1.200	42
**Zn**	2.500–4.000	539
**Hg**	16–25	0.0
**Cr**	0.0	0.0
**Co**	0.0	0.0
**As**	0.0	0.0

**Table 3:** Slagging index regarding Iron to Calcium Ratio and pH of sewage sludge (Sample 1), sewage sludge–soil (Sample 2), and sewage sludge–soil–slag (Sample 3).

Index	Sample 1	Sample 2	Sample 3	Limit Values [21]
**Iron to Calcium Ratio (I/C)** **Formula: Fe_2_O_3_/CaO**	0.667	0.774	0.385	Low: <0.31 or >3 Medium: <10.3 < I/C < 3 High: ≠1
**pH**	7.12	7.03	8.54	

**Table 4:** Polycyclic Aromatic Hydrocarbons (PAH) of sewage sludge (Sample 1), sewage sludge–soil (Sample 2), and sewage sludge–soil–slag (Sample 3).

Polycyclic Aromatic Hydrocarbons [mg/kg su]	Sample 1	Sample 2	Sample 3
**Anthracene**	<0.01	<0.01	<0.01
**-Benzo [a] anthracene**	<0.01	0.01	0.02
**Benzo [b] fluoranthene**	0.01	0.02	0.04
**Benzo [k] fluoranthene**	<0.01	0.01	0.01
**Benzo [g, h, i] perylene**	0.01	0.02	0.04
**-Benzo [a] pyrene**	0.00	0.02	0.03
**-Chrysanthemum**	0.01	0.02	0.02
**-Fluoranthene**	0.01	0.03	0.03
**Indeno [1, 2, 3-cd] pyrene**	0.00	0.00	0.02
**-Naphthalene**	0.00	0.00	0.00
**-Phenanthrene**	0.02	0.02	0.00
**-Pyrene**	0.01	0.03	0.02
**Sum**	**0.07**	**0.18**	**0.23**
**Recommended values from ORDER no. 344/708/2004** [22]	5	5	5

## Data Availability

All data analyzed during this study are included in this published article and its supplementary information files.

## Supplementary Materials

The following supporting information can be downloaded at: https://www.mdpi.com/article/10.3390/ma16196440/s1, Figure S1: EDX spectra—qualitative chemical analysis of the analyzed sewage sludge–soil–slag sample (Sample 3).
