# Supplementary material for: A New Composite Material with Environmental Implications for Sustainable Agriculture
Source: Materials (Basel). 2023 Sep 27;16(19):6440. doi: 10.3390/ma16196440 (PMC10573407; doi:10.3390/ma16196440)
Supplement: Supplementary file 1 [file materials-16-06440-s001.zip › materials-2596113-supplementary.pdf]

A New Composite Material with Environmental Implications for Sustainable Agriculture

Viorica Ghisman [1], Puiu Lucian Georgescu [2], Georgiana Ghisman [1] and Daniela Laura Buruiana [1,*]

[1] Interdisciplinary Research Centre in the Field of Eco-Nano Technology and Advance Materials CC-ITI, Faculty of Engineering, "Dunarea de Jos" University of Galati, 47 Domneasca, 800008 Galati, Romania; viorica.ghisman@ugal.ro (V.G.); georgiana.ghisman@ugal.ro (G.G.)
[2] European Center of Excellence for the Environment, Faculty of Sciences and Environment, University of Galati, 800001 Galati, Romania; lucian.georgescu@ugal.ro
* Correspondence: daniela.buruiana@ugal.ro

The EDX spectra of the raw data of sewage sludge-soil-slag sample (Sample 3) are presented in Figure S1 recorded in different punctual area.

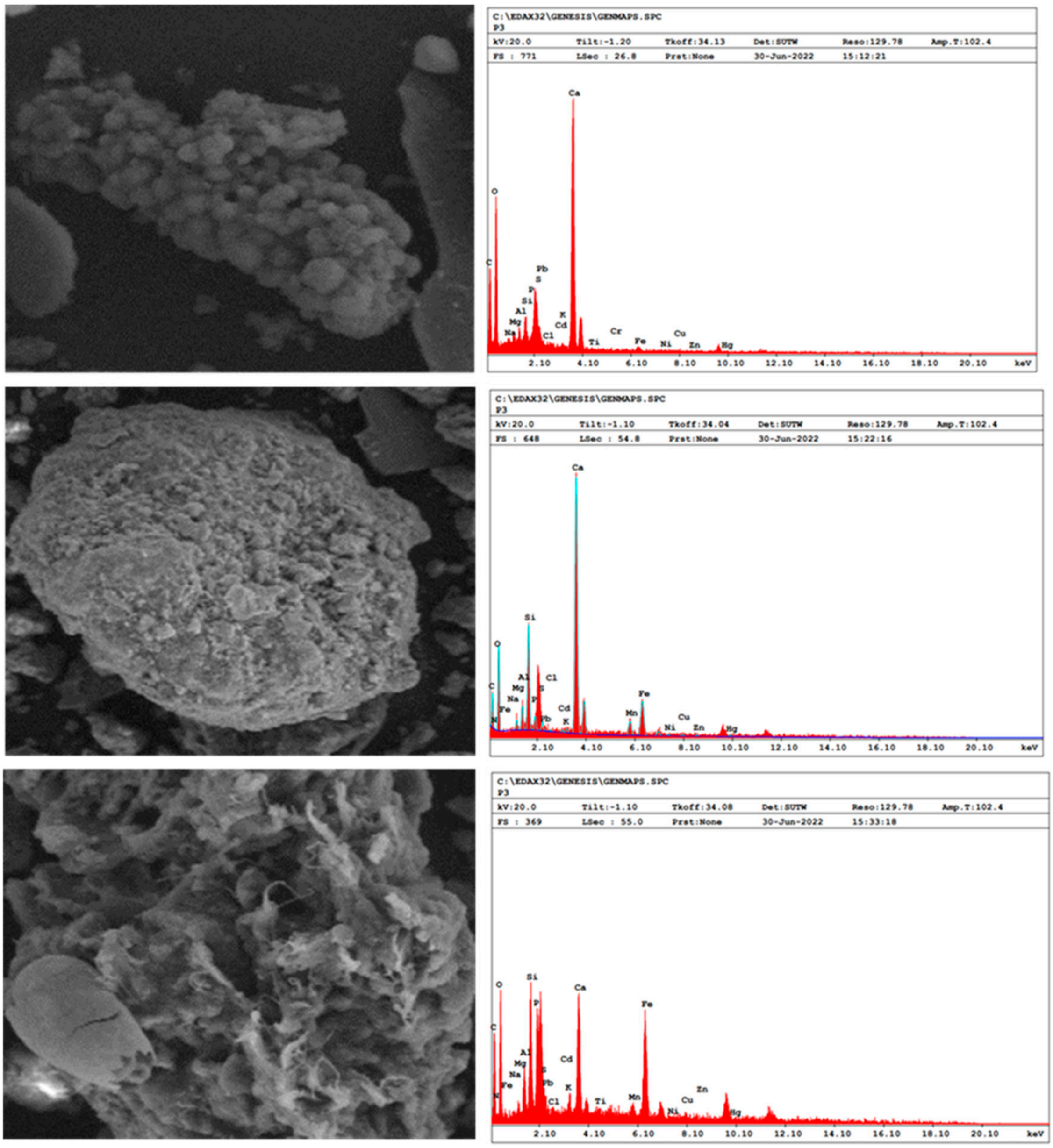

Figure S1: EDX spectra – qualitative chemical analysis of the analysed sewage sludge-soil-slag sample (Sample 3).
